# A Comparative Study on Microbiological and Chemical Characteristics of Small Ruminant Carcasses from Abattoirs in Greece

**DOI:** 10.3390/foods11152370

**Published:** 2022-08-07

**Authors:** Anestis Tsitsos, Vangelis Economou, Eirini Chouliara, Ioannis Ambrosiadis, Georgios Arsenos

**Affiliations:** 1 Laboratory of Hygiene of Food of Animal Origin and Veterinary Public Health, School of Veterinary Medicine, Aristotle University of Thessaloniki, 54124 Thessaloniki, Greece; 2 Laboratory of Technology of Food of Animal Origin, School of Veterinary Medicine, Aristotle University of Thessaloniki, 54124 Thessaloniki, Greece; 3 Laboratory of Animal Husbandry, School of Veterinary Medicine, Aristotle University of Thessaloniki, 54124 Thessaloniki, Greece

**Keywords:** meat quality, abattoir, small ruminant, goat, sheep, *Listeria monocytogenes*, ESBL *Escherichia coli*, chemical composition

## Abstract

Meat quality dictates consumer preferences with hygiene forming a key component, especially in meat types with declining popularity, such as sheep and goat meat. Aiming to increase the marketability of sheep and goat meat, we examined 370 sheep and goat carcasses from two abattoirs in Greece. Tests included enumeration of the total mesophilic viable count, total psychrophilic viable count and coliform count, and detection of *Salmonella* spp., *Listeria monocytogenes* and presumptive ESBL *Escherichia coli*. Moreover, designated samples of meat were used to measure pH, moisture, total fat and protein content. Goat carcasses had significantly higher microbial counts compared to sheep carcasses. Lamb and kid carcasses had larger TMVC, TPVC and coliform counts compared to carcasses from adult animals. One strain of *L. monocytogenes* (0.8%), typed as serovar 1/2a (3a), was isolated from one adult sheep carcass. Twelve strains of ESBL *Escherichia coli* (25%) were isolated; there were not any strains of *Salmonella* spp. The average values of pH, moisture, total fat and total protein were 5.83%, 67.76%, 7.21% and 21.31%, respectively, for sheep carcasses and 5.70%, 68.2%, 5.69% and 24.10%, respectively, for goat carcasses. The results showed a small deviation in assessed parameters, implying the uniformity of the conditions concerning rearing and slaughtering.

## 1. Introduction

Sheep and goat meat are an essential part of the human diet, being an important source of proteins with high biological value, healthy fats and minerals [1,2]. As in most foods, its suitability for human consumption is determined by safety and quality parameters. Considering that the hygiene of food is a prerequisite, quality is subjective to consumer preferences [3,4]. These preferences are affected by intrinsic (i.e., psychological, social, cultural) and extrinsic (i.e., marketing and packaging) factors [3]. Meat quality can be objectively assessed in the lab using certain physicochemical parameters, with the latter not usually perceived by the consumers [1,2]. In terms of sheep and goat meat, there is a large variation in demand and quality depending on production systems and methods of rearing throughout the year, with the latter being subjected to climate and topographical differences [5,6,7]. Thus, the characterization of meat quality relies mostly on empirical characteristics that vary among consumers [6,8].

The microbiological quality of meat is important for both consumer satisfaction and safety. Meat can harbor several saprophytic and pathogenic microorganisms that are likely to deteriorate its quality or endanger the safety of consumers [9]. For example, commensal bacteria such as *Enterococcus* spp., *Pseudomonas* spp., *Psychrobacter* spp., *Acinetobacter* spp., *Aeromonas* spp. and *Moraxella* spp., as well as pathogenic bacteria belonging to the *Staphylococcus*, *Bacillus*, *Campylobacter*, *Clostridium*, *Listeria* and *Salmonella* species, can be found in meat. Other contaminants could be yeasts and molds such as *Candida* spp., *Cryptococcus* spp., *Cladosporium*, *Geotrichum*, *Penicillium* and *Mucor* spp. [9,10]. Considering all the above, it is necessary to control any microbial proliferation and monitor the closely relevant quality parameters of meat to ensure an absence of pathogens and guarantee its safety [11,12,13].

There is abundant evidence in the literature about slaughtering procedures; however, there is scarce information about the microbiological and physicochemical characteristics of sheep and goat carcasses, particularly in Mediterranean countries where the sector is dominated by dairy animals. Most data regarding meat quality are obtained from meat producing sheep and goat breeds. Thus, the main objective of the present study was to assess hygiene and quality of sheep and goat carcasses from abattoirs in Greece.

## 2. Materials and Methods

### 2.1. Abattoirs and Carcasses Evaluated

The study was conducted in two slaughterhouses in Greece from October 2019 to June 2020. The abattoirs were selected based on the number of small ruminants slaughtered annually. Abattoir A is located in Thessaly and abattoir B in Central Macedonia; both are licensed for slaughtering ruminants and pigs with three slaughtering lines (cattle, small ruminants and pigs). The cattle slaughter line is completely separate, whereas the slaughter of small ruminants and pigs is carried out in parallel. Abattoir A is situated in a semi-mountainous area at an altitude of 498 m and has an annual production of approximately 2000 tons of meat. Abattoir B is situated in Central Macedonia at sea-level altitude with an annual production of approximately 2500 tons of meat. The hourly capacity of slaughterhouse A is 10 cattle, 100 pigs and 100 small ruminants, whereas the hourly capacity of slaughterhouse B is 10 cattle, 50 pigs and 150 small ruminants. Both abattoirs have similar practices concerning the slaughter of cattle and small ruminants, with only minor differences. A total of 370 small ruminant carcasses were examined from October 2020 to September 2021; 215 samples were collected from sheep and 155 from goat carcasses. Sheep carcasses were either lambs or adults from fat-tailed dairy sheep breeds (Chios breed) or crosses with other breeds. Goat carcasses were from an indigenous Greek breed or crosses with foreign breeds. Three major age groups were defined; (i) Group A: composed of lambs or kids weighing less than 35% of the mature weight of relative adults, (ii) Group B: composed of sheep and goats weighing between 35% and 70% of the mature weight and (iii) Group C: composed of sheep and goats weighing over 70% of the mature weight. Table 1 shows the numbers of sheep and goat carcasses evaluated, classified by species, gender and age.

The study was conducted according to the guidelines of the Declaration of Helsinki, and approved by the Research Committee of the Aristotle University of Thessaloniki (96813/3 May 2011) within the framework of the GreQuM project.

### 2.2. Microbiological Assay

Each carcass was subjected to surface sampling, approximately 1 h after slaughter, using the non-destructive swab method. In brief, a sterile swab soaked in 5 mL of Maximum Recovery Diluent (MRD, Oxoid Ltd., Basingstoke, UK) and a dry sterile swab were used to wipe a 100 cm^2^ carcass surface area on the hindquarters of sheep and goat carcasses. Then, both swabs were put in a tube containing 5 mL of MRD and transported, in a portable fridge, to the Laboratory of Hygiene of Food of Animal Origin and Veterinary Public Health. Within 24 h, tubes were brought to room temperature, thoroughly vortexed and decimal dilutions were performed in MRD-containing tubes. The sample examination regarded total mesophilic viable counts (TMVCs) and coliform count, and was performed according to ISO 4833/2005 and ISO 21528-2/2017 with modifications, as proposed by the Commission Regulation (EC) No. 2073/2005 on microbiological criteria for foodstuffs. From each dilution, 1 mL was surface-inoculated in appropriate media. Enumeration of TMVCs and total psychrophilic viable counts (TPVCs) was performed in Plate Count Agar (Biolab Diagnostics, Budapest, Hungary). For the coliform count, Violet Red Bile Agar (Biolab Diagnostics, Budapest, Hungary) was used. Incubation was performed at 30 °C for 72 h for TMVCs, 10 °C for 7 days for TPVCs and 37 °C for 24 h for the coliform count. After incubation in appropriate conditions, the characteristic colonies were counted, and results recorded.

Detection of *Salmonella* spp., *Listeria monocytogenes* and presumptive ESBL *Escherichia coli* on the surface of carcasses was carried out following the same non-destructive swab method. In brief, soaked sterile swabs in 50 mL of Buffered Peptone Water (BPW, Oxoid Ltd., Basingstoke, UK) or 50 mL of Half Fraser (HF, Oxoid Ltd., Basingstoke, UK) and dry sterile swabs were used to wipe, in total, a 1000 cm^2^ surface from the forequarter, hindquarter and abdomen area of each carcass. The soaked and the dry swab were put in a tube containing 50 mL of BPW or 50 mL of HF and were transported to the laboratory using a portable fridge. Tubes containing BPW were incubated at 37 °C for 24 h and tubes containing HF were incubated at 30 °C for 18 h.

Examination of samples for the presence of *Salmonella* spp. was performed according to ISO 6579-1/2017 with modifications. In brief, the pre-enrichment of the initial suspension was followed by a selective enrichment on Rappaport–Vassiliadis Broth (RV, Biolab Diagnostics, Budapest, Hungary) at 41.5 °C for 24 h and on Muller–Kauffmann Tetrathionate Broth (TT, Biolab Diagnostics, Budapest, Hungary) at 37 °C for 24 h. Further, 10 μL of inoculums was surface platted on Xylose Lysine Deoxycholate Agar (XLD, Biolab Diagnostics, Budapest, Hungary) and incubated at 37 °C for 24 h. Suspected colonies were verified as *Salmonella* spp. by standard morphological and biochemical characteristics. A total of 123 swab samples were examined for the detection of *Salmonella* spp.

Detection of *L. monocytogenes* in carcass samples was performed according to ISO 11290-1/2017. In brief, 0.1 mL of the initial inoculum in HF was transferred to sterile tubes containing 10 mL of Fraser Broth (Oxoid Ltd., Basingstoke, UK) and incubated at 37 °C for 24–48 h. Then, 10 μL of the inoculum was surface platted on ALOA Agar (Biolab Diagnostics, Budapest, Hungary) and incubated at 37 °C for 24–48 h. Typical colonies of *L. monocytogenes* were purified by surface inoculation on Tryptone Soya Agar with Yeast Extract (TSAYE, Oxoid Ltd., Basingstoke, UK) at 37 °C for 24 h. The pure cultures were subjected to DNA extraction, according to Lawrence and Gilmour [14], by suspending one loopful of cells in 50 μL of sterile Milli-Q water in PCR tubes. The suspension was heated at 100 °C for 10 min, cooled on ice and centrifuged at 13,000 rpm for 5 min. The supernatant was removed from the cell debris, placed into a new sterile tube and stored at −20 °C. The extracted samples were subjected to a multiplex PCR for the molecular identification and the molecular serotyping of *L. monocytogenes*, according to the protocol of Doumith et al. [15] with modifications. PCR was performed with a 25 μL volume containing 2U of OneTaq™ DNA Polymerase (M0273S, NEB), 2.5 μL of 10× OneTaq Standard Reaction Buffer (NEB), 200 μM of dNTPs (N0447S, NEB), 0.25–2.5 μL of primers and 2 μL of sample DNA (Table 2). PCR was carried out on a thermal cycler (LabCycler Gradient, SensoQuest GmbH, Göttingen, Germany) with the initial denaturation (180 s at 94 °C) followed by 35 cycles of amplification (denaturation at 94 °C for 24 s, annealing at 53 °C for 69 s, and extension at 72 °C for 69 s) and ending with a final extension at 72 °C for 7 min. The PCR products were detected by electrophoresis in 1.5% agarose gels stained with ethidium bromide. A total of 123 swab samples were examined for the presence of *L. monocytogenes*.

For the isolation of presumptive ESBL *E. coli*, the protocol of the EFSA Panel on Biological Hazards was used, with modifications [16]. In brief, 10 μL of the initial inoculum in BPW was surface platted in TBX Agar (Biolab Diagnostics, Budapest, Hungary) containing 1 mg/L of cefotaxime and incubated at 42 °C for 24 h. Typical colonies were phenotypically confirmed by the standard disk diffusion [17]. A total of 48 swab samples were examined for the isolation of ESBL *E. coli* from the surface of sheep and goat carcasses.

### 2.3. Physicochemical Characterization

Meat samples were also collected from cold carcasses to assess physicochemical characteristics. In brief, meat samples weighing approximately 100 g were obtained from the muscle *Quadriceps femoris* and transported to the laboratory the same day, where they were comminuted with a Waring laboratory blender. The samples were used to measure pH, moisture, total fats and total proteins. For pH measurement, 10 g of muscle was dispersed in 40 mL of distilled water and left to settle. The analysis of pH was performed with a Hanna Ph211 568 pH Meter. For moisture measurement, 3 g of meat was weighed and placed on an aluminum sample pan. A moisture analyzer (MB27, Ohaus, Parsippany, NJ, USA), at 105 °C, was used according to the manufacturer’s instructions. Total fat was determined with the Weibull–Stoldt method; hydrolysis was the first step, which was followed by using the Soxtherm Soxhlet Extraction System, according to AOAC Method 991.36. The protein of the sample was determined according to AOAC Official Method 928.08. A total of 58 sheep and goat samples were examined for their physicochemical properties.

### 2.4. Statistical Analysis

Both parametric and non-parametric statistical methods were applied for the statistical evaluation of results. Parameters were assessed using measures of central tendency and dispersion to reveal the characteristics of the sample. All analyses were conducted using the statistical software program IBM SPSS Statistics (v.27.0., IBM Corporation, Armonk, NY, USA). Significance was set at *p*-value ≤ 0.05, unless otherwise noted.

## 3. Results

### 3.1. Microbiological Results

**Figure 1:**
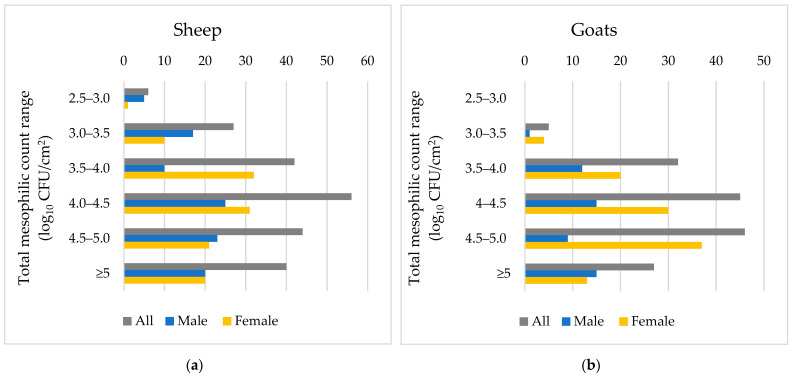
Number of sheep (**a**) and goat (**b**) carcasses categorized according to total mesophilic count (log_10_ CFU/cm^2^) range.

Microbial counts in goat carcasses according to species, gender and age are shown in Table 3. TMVCs were 3.76 log_10_ CFU/cm^2^ and 3.92 log_10_ CFU/cm^2^, TPVCs were 2.97 log_10_ CFU/cm^2^ and 3.32 log_10_ CFU/cm^2^, and coliform counts were 1.8 log_10_ CFU/cm^2^ and 2.15 log_10_ CFU/cm^2^ in sheep and goat carcasses, respectively. Goat carcasses had significantly higher microbial counts compared to sheep carcasses, as shown in Figure 1. Moreover, lamb and kid carcasses, in Group A, had more TMVCs, TPVCs and coliform counts compared to Group C. Differences in microbial counts among age groups were greater in goats than sheep (Figure 1). Microbial counts in carcasses across gender were similar with no significant differences. One strain of *Listeria monocytogenes* (0.8%) was isolated from one adult sheep carcass, whereas 12 strains of ESBL *Escherichia coli* (25%) were isolated from carcasses of all age groups. The *Listeria monocytogenes* isolate was further typed as serovar 1/2a (3a). No strains of *Salmonella* spp. were isolated.

**Figure 2:**
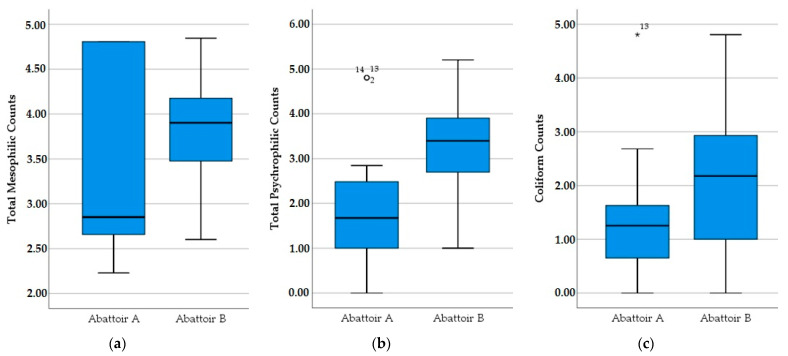
Total mesophilic, total psychrophilic and coliform counts (in log_10_ CFU/cm^2^) of sheep and goat carcasses in the two abattoirs. * The star value is generally accepted as values exceeding the quartetiles. (**a**) Total Mesophilic Counts, (**b**) Total Psychrophilic Counts, (**c**) Coliform Counts.

The average values recorded in samples from Abattoir A and Abattoir B were 3.53 log_10_ CFU/cm^2^ and 3.87 log_10_ CFU/cm^2^ for TMVCs, and 1.81 log_10_ CFU/cm^2^ and 3.29 log_10_ CFU/cm^2^ for TPVCs, whereas the coliform count was 1.19 log_10_ CFU/cm^2^ and 2.05 log_10_ CFU/cm^2^, respectively. In Abattoir B, significant differences were found in TMVCs, TPVCs and coliform counts compared to Abattoir A. There was a larger variation of TMVCs in abattoir A compared to abattoir B; however, the median was larger in Abattoir B. Figure 2 shows boxplot graphs of microbial counts under investigation in the two abattoirs.

### 3.2. Physicochemical Results

**Figure 3:**
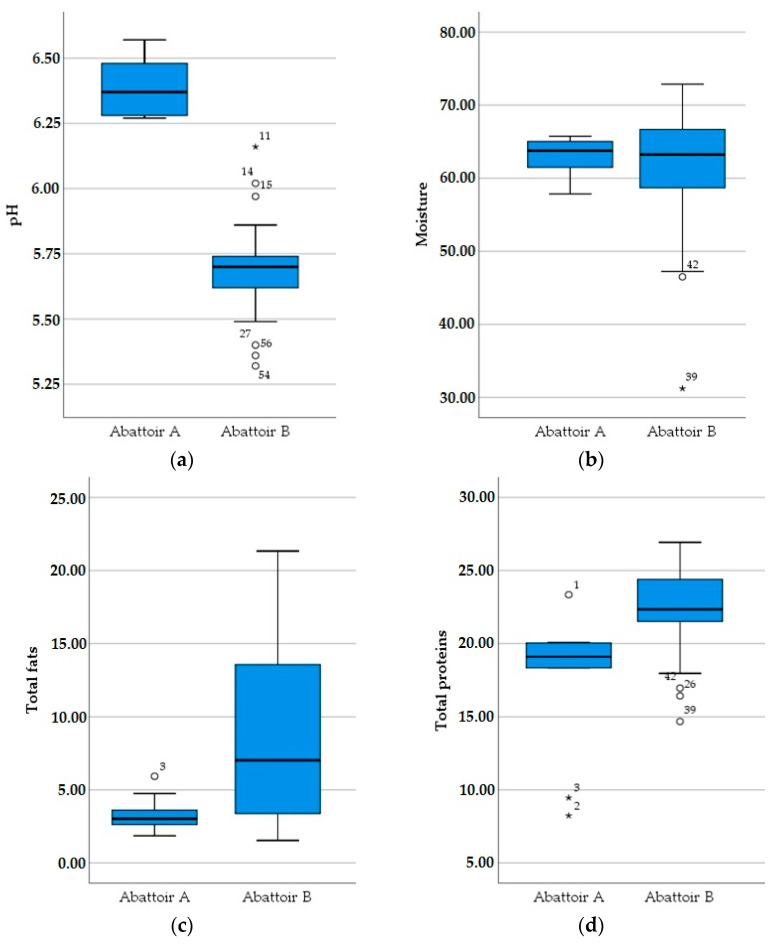
Meat pH (**a**), moisture (**b**), total fats (**c**) and total proteins (**d**) from the two abattoirs under investigation. * The star value is generally accepted as values exceeding the quartetiles.

Concerning the physicochemical analyses of samples from sheep and goat hindquarters, the average pH was 5.83 and 5.7, moisture was 67.76% and 68.2%, total fat was 7.21% and 5.69%, and total proteins were 21.31% and 24.10%, respectively. Goat carcasses had a significantly higher concentration of total proteins compared to sheep carcasses. There were no significant differences among the carcasses of different genders and age groups. The moisture of the cold carcasses was similar in the two abattoirs. However, there were significant differences in pH, total fat and total proteins of samples from different abattoirs, as shown in Figure 3. Results regarding physicochemical analyses are reported in Table 4.

## 4. Discussion

As asserted in the Introduction section, the objective was to assess the hygienic status and quality of sheep and goat meat produced in two Greek abattoirs. The results showed that microbial counts were generally above the limits set by the EU Commission Regulation No. 2073/2005 concerning microbiological criteria for foodstuffs. In some carcasses, microbial counts exceeded the average limit of 3.5 log_10_ CFU/cm^2^, but were below the upper limit of 5.0 log_10_ CFU/cm^2^ TMVC. According to Bhandare et al. [18], the total aerobic bacteria count of sheep carcasses slaughtered in Indian abattoirs was, on average, 5.13 ± 0.36 log_10_ CFU/cm^2^, whereas the coliform count was 3.31 ± 0.19 log_10_ CFU/cm^2^. Tanganyika et al. [19] reported that the TMVCs of goat carcasses in Malawi ranged from 5.75 log_10_ CFU/cm^2^ to 8.22 log_10_ CFU/cm^2^. In this study, we found lower values that are likely related to better hygienic conditions, washing and evisceration techniques in Greek abattoirs. Yalçin et al. [20] examined the surface contamination in carcasses of sheep slaughtered in a Turkish abattoir. They reported that mean TMVCs and total coliforms after the washing step were 2.8 log_10_ CFU/cm^2^ and 0.84 log_10_ CFU/cm^2^, respectively; both were lower compared to the ones in this study. Zweifel and Stephan [21] report that the contamination rates for TMVCs in three Swiss abattoirs ranged from 2.5 log_10_ CFU/cm^2^ to 3.8 log_10_ CFU/cm^2^, which are comparable with the ones observed in the present study. Still, it should be noted that the comparison of microbial contamination of carcasses with data from countries outside the European Union is problematic, since in other areas and, more specifically, in North America, they are hampered by the application of decontamination procedures not in use in European abattoirs [22,23].

Sierra et al. [24] reported larger microbial counts after the washing step in a sheep abattoir in Ireland; the average microbial counts after washing in the four plants investigated ranged from 4.63 to 4.88 log_10_ CFU/cm^2^. The larger counts can be attributed to different carcass areas being sampled, such as the abdomen. The abdomen can be contaminated during the evisceration; this stage is considered the most implicated in carcass contamination by Enterobacteriaceae. Still, no correlation was observed between the total counts and the Enterobacteriaceae in this study. Milios et al. [25] reported that TMVCs and Enterobacteriaceae counts in a Greek lamb abattoir were 5.89 and 3.74 log_10_ CFU/cm^2^, respectively. They also proposed that steam decontamination could greatly benefit the overall microbial quality of the sheep carcass. Røssvol et al. [26] compared the effects of two evisceration methods on the hygiene of sheep carcasses, stating that no difference existed between them.

In this study, significant differences regarding TMVCs, TPVCs and coliform counts were observed between the two abattoirs. In Abattoir B, TMVCs, TPVCs and coliform counts were higher compared to Abattoir A. The latter raised concerns regarding slaughtering procedures in Abattoir B; however, its procedures resulted in less contaminated carcasses overall. Salmela et al. [27] examined the microbial contamination of sheep carcasses in four abattoirs in Finland. Significant differences were reported among the slaughterhouses in TVMCs, but not in total coliforms, indicating the different hygiene measures implemented amongst abattoirs. Similarly, Bhandare et al. [18] reported differences in TVMCs of sheep and goat carcasses between modern abattoirs and traditional meat shops in India, but no differences were observed in total coliforms. Zweifel and Stephan [21] examined the microbiological contamination of sheep carcasses in three Swiss abattoirs. They reported few differences in the microbial counts among the slaughterhouses. However, significant differences in TMVCs and total coliforms were observed on different slaughtering days and different sites of the carcass. In this study, goat carcasses had larger TMVCs, TPVCs and coliform counts compared to sheep carcasses. Carcasses from lambs and kids had larger microbial counts compared to those of adult animals, most likely due to inadequate slaughtering procedures. The latter findings are in accordance with the results of Kim et al. [28], who observed significantly higher TMVCs in goat meat purchased from retail markets than those of lamb meat. However, Ahmad et al. [29] reported that TMVCs of sheep and goat meat isolated from abattoirs of Lahore were not statistically significant. Differences observed in the microbial counts may also be explained by differences in foraging behavior and diet selection of sheep and goats [28,30].

The results in this study revealed an absence of *Salmonella* spp., whereas one strain of *Listeria monocytogenes* serotype 1/2a (3a) and 12 strains of ESBL *E. coli* were isolated. Regarding *Salmonella* spp., the results confirm the findings of previous studies [18,27,31], but are also contradictory to others, such as Ahmad et al. [29] that reported *Salmonella* spp. isolation from 10% of sheep and goat carcasses in slaughterhouses of Lahore. *Salmonella* Typhimurium, *Listeria ivanovii* and *Listeria innocua* were also isolated from air samples in a sheep abattoir in Ireland. In this study, no strains of *L. monocytogenes* were isolated, whereas the bacterial counts were generally low, suggesting that the aerial counts of *Salmonella* spp. and *Listeria* spp. are unlikely to be a significant source of carcass contamination [32]. Ayaz et al. [11] isolated seven strains of *L. monocytogenes* serovar 1/2a (3a) from 3 sheep carcasses in Turkey out of the 120 examined (2.5%), indicating a slightly higher prevalence of *L. monocytogenes* in sheep carcasses compared to this study. Lotfollahi et al. [33] reported the isolation of three strains of *L. monocytogenes* from sheep and goat carcasses in Iran, two of which belonged to serovar 4b, 4d or 4e, and one to 1/2a or 3a. Generally, *L. monocytogenes* serotype 4b is responsible for up to 50% of human listeriosis cases globally, whereas serotype 1/2a is more frequently isolated from food products [34]. Regarding ESBL *E. coli*, Atlaw et al. [35] reported that the prevalence of ESBL *E. coli* isolated from sheep carcasses in the U.S. was 10.2%, which is lower compared to the prevalence in this study. The lower prevalence may be explained by the sanitary dressing procedures followed in U.S. abattoirs and the application of lactic acid carcass decontamination usually performed [13,22,23]. Similarly, Bhoomika et al. [12] reported a lower prevalence of ESBL *E. coli* in goat carcasses in India, as they isolated 7 ESBL *E. coli* strains out of 82 samples examined (18.42%). On the contrary, the prevalence of ESBL *E. coli* in retail sheep meat from Brazil was 60% [36], although it should be noted that the prevalence may be inaccurate due to the limited number of samples tested (*n* = 25).

Considering the results of pH, moisture, total fat and total proteins in meat samples examined in this study, the variation between average measurements was relatively small, implying the repeatability of slaughtering procedures and uniformity of conditions in the whole process. The observed variation in total fat concentrations of sheep carcasses can be attributed to the number of samples examined and the inclusion of samples from mature animals that are generally fatter [2].

The results showed that pH values of sheep meat declined faster compared to goat meat. In the study of Shija et al. [37], the average pH of goat carcasses at 24 h was higher compared to those of sheep, being 5.88 and 5.74, respectively. This may be explained by possible differences in the ante mortem stress of animals. Katsaounis et al. [38] examined the average meat composition of lamb carcasses of Karagouniko, Serres and Boutsko breeds. The total proteins were 14.8% (12.1–17.5%), total fats were 30.3% (16.2–44.4%) and moisture was 50.85% (40.2–61.5%). The results of moisture and total proteins are lower compared to those of this study, but total fats are significantly higher, possibly due to the fact that they were slaughtered at different stages of development. Arsenos et al. [39] reported that goat meat from Greek domestic breeds had a pH value of 5.61–5.63, a water percentage of 74.2%, total proteins of 19.9% and total fats of 4.9%. Apart from moisture, these values are lower compared to the ones presented in this study.

In the present study, cold goat carcasses had a significantly higher concentration of total proteins compared to cold sheep carcasses. However, apart from total proteins, no differences were observed in the moisture and total fats between sheep and goat carcasses. This is in accordance with the results of Lee et al. [40], who found no significant differences in moisture and fat contents between sheep and goats. Okuskhanova et al. [41] reported a higher concentration of total proteins in goat carcasses, but also a higher moisture content and a lower fat content. Shija et al. [37] found statistically significant differences in moisture and total fats of sheep and goat carcasses, but not in total proteins. Moreover, significant differences were also observed between the different abattoirs, even within the same animal species, in all physicochemical characteristics of the carcasses apart from moisture. These differences can be explained by the different characteristics of animals slaughtered in each abattoir.

Meat quality can be affected by various intrinsic and extrinsic factors, such as species, breed, sex, age and nutrition [2,42]. These factors are difficult to balance between studies, and hence, result in debate between authors regarding the chemical composition of sheep and goat carcasses. In the present study, small differences were observed among carcasses of the same species, but with different gender and age groups. The evidence in the literature suggests that total proteins and total fats increase with the age of sheep and goats, whereas moisture content decreases [43,44]. On the contrary, Junkuszew et al. [45] reported a lower concentration of total proteins in adult sheep meat compared to lamb meat. Madruga et al. [46] stated that the sex of sheep did not significantly affect the chemical composition of meat. However, the contents of phosphorus and calcium, as well as the fatty acid profile of sheep meat, were significantly affected by the sex.

The two abattoirs involved in this study are situated in different areas, with Abattoir A being in a semi-mountainous area and Abattoir B being in a lowland area. Concerning the physicochemical parameters examined, differences were observed in the pH, the total fat concentration and the total protein concentration. No differences were observed to the moisture content. The abattoirs in Greece usually slaughter small ruminants within their vicinity; therefore, it is highly likely that animals slaughtered in these abattoirs originate from the semi-mountainous area and lowland area where Abattoir A and Abattoir B are situated. Grazing animals are reported to suffer from nutritional imbalance, at least during the grazing period of the year, with a direct effect on their protein and energy metabolism [47]. In the study of Ådnøy et al. [48], the quality of meat produced by sheep reared in different altitudes was compared. It is reported that the fat content of meat derived from lowland-raised animals was higher than that of the mountain raised; still, the meat protein concentration was similar in both areas. Usually, small ruminants in semi-mountainous areas tend to graze for larger periods than in lowland areas. It is quite probable that this difference in feeding has resulted in differences in the chemical composition of meat, as reported elsewhere [49,50].

## 5. Conclusions

In this study, the results of microbiological and physicochemical analyses of 370 carcasses from different abattoirs in Greece were reported. The microbial counts examined show that the hygiene of meat production at the abattoir stage needs to be improved, since in some cases the microbial counts were above the limits posed by the Commission Regulation (EC) No. 2073/2005. Goat carcasses especially showed larger microbial counts compared to sheep carcasses, whereas the carcasses of younger animals had larger microbial counts compared to mature animals. The prevalence of pathogens was low, with no *Salmonella* spp. isolated, whereas *Listeria monocytogenes* and ESBL *Escherichia coli* were isolated from 0.8% and 25% of the carcasses sampled, respectively. Differences in the chemical composition of meat from different abattoirs, attributed to the rearing area, were observed. The results revealed the need for further research to monitor the microbiological quality and hygiene of meat produced in Greek slaughterhouses.

## Figures and Tables

**Table 1:** Examined number of sample carcasses according to species, gender and age.

Species	Gender		Age			Total
	Male	Female	<35%	35–70%	>70%	
Sheep	100	115	86	36	93	215
Goat	51	104	51	21	83	155
Total	151	219	137	57	176	370

**Table 2:** Primer list, desired concentration and predicted sizes of PCR products for the molecular identification and molecular serotyping of *Listeria monocytogenes*.

Gene	Primers	Product (bp)	Target
*prs*	For: GCTGAAGAGATTGCGAAAGAAG	370	All *Listeria* species
	Rev: CAAAGAAACCTTGGATTTGCG		
*ORF2819*	For: AGCAAAATGCCAAAACTCGT	471	Serovars 1/2b, 3b, 4b, 4d and 4e
	Rev: CATCACTAAAGCCTCCCATTG		
*ORF2110*	For: AGTGGACAATTGATTGGTGAA	597	Serovars 4b, 4d and 4e
	Rev: CATCCATCCCTTACTTTGGAC		
*lmo0737*	For: AGGGCTTCAAGGACTTACCC	691	Serovars 1/2a, 1/2c, 3a and 3c
	Rev: ACGATTTCTGCTTGCCATTC		
*lmo1118*	For: AGGGGTCTTAAATCCTGGAA	906	Serovars 1/2c and 3c
	Rev: CGGCTTGTTCGGCATACTTA		

**Table 3:** Microbial counts of sheep and goat carcasses (in log_10_ CFU/cm^2^), based on gender and age (average in bold, standard deviation in parentheses and italics).

			TMVC	TPVC	Coliforms
Sheep	Gender	Male	**3.75** (*0.76*)	**2.92** (*1.24*)	**2.08** (*1.27*)
		Female	**3.77** (*0.63*)	**3.01** (*0.78*)	**1.56** (*1.27*)
	Age	<35%	**3.75** (*0.82*)	**2.66** (*1.13*)	**1.89** (*1.22*)
		35–70%	**3.63** (*0.63*)	**3.04** (*0.96*)	**2.04** (*1.11*)
		>70%	**3.81** (*0.58*)	**3.22** (*0.85*)	**1.64** (*1.42*)
	Total		**3.76** (*0.69*)	**2.97** (*1.02*)	**1.80** (*1.30*)
Goat	Gender	Male	**3.97** (*0.60*)	**3.12** (*1.05*)	**2.66** (*1.44*)
		Female	**3.90** (*0.52*)	**3.42** (*0.86*)	**1.90** (*1.20*)
	Age	<35%	**4.17** (*0.52*)	**3.63** (*0.92*)	**3.17** (*1.21*)
		35–70%	**4.13** (*0.33*)	**3.72** (*0.48*)	**2.43** (*0.77*)
		>70%	**3.72** (*0.53*)	**3.03** (*0.94*)	**1.45** (*1.45*)
	Total		**3.92** (*0.55*)	**3.32** (*0.94*)	**2.15** (*1.33*)

**Table 4:** Physicochemical analyses of sheep and goat carcasses, based on gender and age (average in bold, standard deviation in parentheses and italics).

			pH	Moisture	Total Fat	Total Proteins
Sheep	Gender	Male	**5.89** (*0.38*)	**63.27%** (*4.78*)	**5.98%** (*4.5*)	**20.73%** (*4.39*)
		Female	**5.77** (*0.24*)	**60.19%** (*9.40*)	**8.50%** (*6.46*)	**21.92%** (*3.32*)
	Age	<35%	**5.86** (*0.33*)	**62.75%** (*4.98*)	**6.41%** (*4.93*)	**20.22%** (*4.53*)
		35–70%	**5.95** (*0.41*)	**60.85%** (*3.96*)	**8.10%** (*6.05*)	**21.25%** (*3.16*)
		>70%	**5.74** (*0.25*)	**61.15%** (*10.56*)	**7.63%** (*6.32*)	**22.48%** (*3.32*)
	Total		**5.83** (*0.32*)	**61.76%** (*7.48*)	**7.21%** (*5.63*)	**21.31%** (*3.90*)
Goat	Gender	Male	**5.71** (*0.10*)	**63.27%** (*3.59*)	**5.59%** (*4.12*)	**24.29%** (*2.72*)
		Female	**5.68** (*0.15*)	**64.60%** (*2.41*)	**5.83%** (*2.17*)	**23.82%** (*1.73*)
	Age	<35%	**5.67** (*0.12*)	**64.49%** (*3.34*)	**5.36%** (*4.11*)	**24.68%** (*2.47*)
		35–70%	**5.70** (*0.12*)	**63.75%** (*3.10*)	**5.68%** (*3.22*)	**24.05%** (*2.26*)
		>70%	**5.73** (*0.10*)	**62.77%** (*2.78*)	**6.17%** (*2.11*)	**23.22%** (*1.13*)
	Total		**5.70** (*0.11*)	**63.80%** (*3.14*)	**5.96%** (*3.38*)	**24.10%** (*2.12*)

## Data Availability

The data presented in this study are available on request from the corresponding author.
